# Integration of high-throughput reporter assays identify a critical enhancer of the *Ikzf1* gene

**DOI:** 10.1371/journal.pone.0233191

**Published:** 2020-05-26

**Authors:** Jaafar Alomairi, Anne M. Molitor, Nori Sadouni, Saadat Hussain, Magali Torres, Wiam Saadi, Lan T. M. Dao, Guillaume Charbonnier, David Santiago-Algarra, Jean Christophe Andrau, Denis Puthier, Tom Sexton, Salvatore Spicuglia

**Affiliations:** 1 Aix-Marseille University, Inserm, TAGC, UMR1090, Marseille, France; 2 Equipe Labélisée Ligue Contre le Cancer, Marseille, France; 3 Institute of Genetics and Molecular and Cellular Biology (IGBMC), Illkirch, France; 4 CNRS UMR7104, Illkirch, France; 5 INSERM U1258, Illkirch, France; 6 University of Strasbourg, Illkirch, France; 7 Institut de Génétique Moléculaire de Montpellier, Univ Montpellier, CNRS, Montpellier, France; University of Crete & IMBB-FORTH, GREECE

## Abstract

The *Ikzf1* locus encodes the lymphoid specific transcription factor Ikaros, which plays an essential role in both T and B cell differentiation, while deregulation or mutation of IKZF1/*Ikzf1* is involved in leukemia. Tissue-specific and cell identity genes are usually associated with clusters of enhancers, also called super-enhancers, which are believed to ensure proper regulation of gene expression throughout cell development and differentiation. Several potential regulatory regions have been identified in close proximity of *Ikzf1*, however, the full extent of the regulatory landscape of the *Ikzf1* locus is not yet established. In this study, we combined epigenomics and transcription factor binding along with high-throughput enhancer assay and 4C-seq to prioritize an enhancer element located 120 kb upstream of the *Ikzf1* gene. We found that deletion of the E120 enhancer resulted in a significant reduction of *Ikzf1* mRNA. However, the epigenetic landscape and 3D topology of the locus were only slightly affected, highlighting the complexity of the regulatory landscape regulating the *Ikzf1* locus.

## Introduction

Cell-type specific regulation of gene expression requires the activation of promoters by distal genomic elements defined as enhancers. The classical view of enhancer function is that they contribute to increasing the overall level of gene expression by inducing transcription from associated promoters [1]. Complex gene regulation is mediated by the association of clusters of enhancers, also called super-enhancers [2]. Whether the individual components (i.e. single enhancers) synergistically contribute to transcription regulation of their target genes or have distinct specialized functions has been a matter of debate [2–5].

With the increasing awareness of the important role of enhancers in normal development as well as in disease, there is strong scientific interest in identifying and characterizing these elements. However, few predicted enhancer elements have been shown to affect transcription of their endogenous genes or to alter phenotypes when disrupted, highlighting the need to integrate different epigenomic resources and functional assays to identify critical distal regulatory elements [6]. Although putative enhancers can be identified genome-wide based on chromatin accessibility or histone modifications [7], these approaches do not provide direct proof of enhancer function. Recent developments of functional high-throughput assays have enabled quantitative measurements of enhancer activity of thousands of regulatory elements in parallel, providing a straightforward approach to prioritize *bona fide* enhancers [8]. In particular, a common observation of high-throughput assays based on massively paralleled reporter assays [9–14] or CRISPR-based screens [15, 16] is that many predicted enhancer regions do not show enhancer activity in reporter assays or after CRISPR deletion. Therefore, it is crucial to experimentally assess whether genomic regions function as *bona fide* enhancers in living cells.

Ikaros is a lymphoid specific transcription factor that plays a major role in both T and B cell differentiation [17, 18]. During T cell differentiation Ikaros is required for proper gene regulation during the CD4^-^CD8^-^ (double-negative; DN) to the CD4^+^CD8^+^ (double-positive; DP) transition (also called b-selection) mainly by recruiting chromatin repressors [19, 20] and silencing Notch1 target genes [20–23]. Importantly, Ikaros deregulation or mutation plays an important role in leukemia [24–33]. In mouse and human, Ikaros is encoded by the *Ikzf1* gene and is known to harbor several transcript isoforms playing different regulatory roles [34–38]. Several potential regulatory regions have been identified in the proximity of the *Ikzf1* locus, suggesting a complex network of regulatory elements are required to drive Ikaros expression during hematopoiesis and lymphocyte maturation [39–41]. To gain insight into the regulation of *Ikzf1* locus in T cell precursors, we have integrated data from high-throughput reporter assays, chromatin modifications, binding of key T cell transcription factors as well as genomic interactions. We prioritized an enhancer located 120 kb upstream of *Ikzf1* and studied the functional role of this regulatory element.

## Material and methods

### Cell culture

P5424 cell line [42] was kindly provided by Dr. Eugene Oltz, Washington, USA and was cultured as described previously [14]. Cells were passed every 2–3 days and routinely tested for mycoplasma contamination, and maintained in RPMI medium (Gibco) supplemented with 10% FBS (Gold, PAA) at 37 °C, 5% CO2. J1 mouse embryonic stem (ES) cells were grown on gamma-irradiated mouse embryonic fibroblast cells under standard conditions (4.5 g/L glucose-DMEN, 15% FCS, 0.1 mM non-essential amino acids, 0.1 mM beta-mercaptoethanol, 1 mM glutamine, 500 U/mL LIF, gentamicin), then passaged onto feeder-free 0.2% gelatin-coated plates for at least two passages to remove feeder cells before 4C.

### Isolation of DN3 thymocytes

Thymuses from 4–5 weeks old c57/Bl6 mice were dissected and homogenized in cold PBE (PBS with 0.5% BSA and 2 mM EDTA), before incubation for 30 min at 4 °C with rat anti-CD4 and rat anti-CD8 anti-sera (gift from Susan Chan) and depletion of DP thymocytes with sheep anti-rat IgG magnetic beads (Invitrogen). 1x10^8^ cells were resuspended in 300 μl PBE plus 3 μl anti-mouse CD4-FITC, 3 μl anti-mouse CD8a-FITC, 3 μl anti-mouse CD3e-FITC, 3 μl anti-mouse B220-FITC, 3 μl anti-mouse CD11b-FITC, 3 μl anti-mouse Ly-6G(Gr-1)-FITC, 3 μl anti-mouse NK1.1-FITC, 6 μl anti-mouse CD44-PE, and 6 μl anti-mouse CD25-PE (all eBioscience antibodies), and incubated on ice for 10 min before washing in PBE. DAPI was added to a final concentration of 100 ng/ml and live DN3 cells were purified by FACS (DAPI-negative, FITC-negative, APC-negative, PE-positive) before immediate fixation for 4C.

### CRISPR/Cas9 genome editing

The targeted enhancer regions were defined by the peaks of CapStarr-seq and DNase-seq which bind the 6 TFs. Two gRNAs were designed at each end of the targeted region by CRISPR direct tool [43]. The gRNAs were cloned into the gRNA cloning vector (Addgene #41824) as previously described [44]. Two million cells were transfected with 3μg of each gRNA vector and 3μg of Cas9 vector (Addgene #41815) using the Neon transfection system (Life Technologies). After 3 days of transfection, the bulk transfected cells were plated in 96-well plates at the limiting dilution (0.5 cell per 100 μl per well) for clonal expansion. After 10–14 days, individual cell clones were screened for homozygous allele deletion by direct PCR using Phire Tissue Direct PCR Master Mix (Thermo Scientific) following the manufacturer’s protocol. Forward and reverse primers were designed bracketing the targeted regions allowing the detection of knockout and wild-type alleles. Clones with homologous allele deletion were considered if having at least one expected deletion band and no wild-type band. The gRNAs and primers are listed in the S1 Table.

### Gene expression analysis

Total RNA was isolated using the RNeasy kit (Qiagen). RNA samples (1 μg) were reverse-transcribed into cDNA using Superscript VILO Master Mix (Thermo Scientific). The quantitative PCR was performed using power SYBR Master Mix (Thermo Scientific) on a QuantStudio 6 Flex Real-Time PCR System. Primer sequences are listed in S2 Table. Gene expression was normalized to that of *Rpl32*. The relative expression was calculated by delta Ct method and all the shown data reported from the fold change over the control. For each cell clone, the Student’s *t*-test was performed (unpaired, two-tailed, 95% confidence interval) from 3 biological replicates of independent cDNA preparations. Data are represented with standard deviation (s.d). For RT-qPCR, 1/20 of synthesized cDNA was used as template for one reaction; PCRs were performed with Phusion polymerase (Thermo Scientific), Tm = 60 °C, 35 cycles. RNA-seq from the P5424 cell line treated with either DMSO or PMA/ionomycin was published before [45] and was retrieved from GEO (GSE120655).

### PMA/ionomycin induction

1 x 10^6^ cell/ ml of P5424 cells (wt and ΔIkE120) were stimulated for 6 hours with 20 μg/ml of PMA plus 0.5 μg/ml of ionomycin in 6-well plate of 3 independent experiments. Then, total RNA was prepared from non-stimulated or stimulated cells using the RNeasy kit (Qiagen) as recommended by the manufacturer.

### Chromatin immunoprecipitation-sequencing (ChIP-seq)

Total 40 x10^6^ of wt and ΔIkE120P5424 cells were crosslinked in 1% formaldehyde for 10 min at 20 °C, followed by quenching with glycine at a final concentration of 250 mM. Pelleted cells were washed twice with ice-cold PBS, and then re-suspended in lysis buffer (20 mM Hepes PH 7.6, 1% SDS, 1X PIC) at final cell concentration of 15 x 10^6^ cells/ml. Chromatin was sonicated with Bioruptor (Diagenode) to an average length of 200–400 bp (5 pulses of 30 sec ON and 30 sec OFF). An aliquot of sonicated cell lysate equivalent to 0.5 x 10^6^ cells was diluted with SDS-free dilution buffer (1% Triton X-100, 1.2 mM EDTA, 16.7 mM Tris pH 8.0, 167 mM NaCl) for single immunoprecipitation. Specific antibody to H3K27ac (C15410196; Diagenode) (1 μg per ChIP) and proteinase inhibitor cocktail were added to the lysate and rotated overnight at 4 °C. On the next day, Protein A magnetic beads (Invitrogen) were washed twice with dilution buffer (0.15% SDS, 1% Triton X-100,1.2 mM EDTA, 16.7 mM Tris pH 8.0, 167 mM NaCl and 0.1% BSA) and added to the lysate and rotated 1 hour at 4 °C. Then, beads were washed with each of the following buffers: once with Wash Buffer 1 (2 mM EDTA, 20 mM Tris pH 8.0, 1% Triton X-100, 01% SDS, 150 mM NaCl), twice with Wash Buffer 2 (2 mM EDTA, 20 mM Tris pH 8.0, 1% Triton X-100, 0.1% SDS, 500 mM NaCl), twice with Wash Buffer 3 (1 mM EDTA, 10 mM Tris pH 8.0). Finally, beads were eluted in Elution buffer (1% SDS, 0.1 M NaHCo_3_) and rotated at RT for 20 min. Eluted materials were then added with 0.2 M NaCl, 0.1 mg/ml of proteinase K and incubated overnight at 65 °C reverse cross-linking, along with the untreated input (10% of the starting material). The next day, DNA was purified with QIAquick PCR Purification Kit (Qiagen) and eluted in 30 μl of water. At least 1 ng of ChIP was used for library preparation. Libraries for ChIPs against H3K27ac was prepared according to Illumina ChIP-Seq protocol and sequenced on a Nextseq500 (Illumina) according to the manufacturer’s instructions. ChIP-seq was processed as described previously [14] and RPKM-normalized using deepTools bamCoverage [46] before visualization as heatmaps with deepTools plotHeatmap and as genome tracks with the IGV genome browser [47]. Differential H3K27ac signal between wt and ΔIkE120 cells was generated with the IGV genome browser.

### CapStarr-seq

CapStarr-seq data in P5424 (two replicates) and NIH3T3 cell lines with a selected set of DHSs were previously published [14] and processed data retrieved from GEO (GSE60029). As described previously, the enhancer activity was computed by calculating the ratio (fold change; FC) of FPKMs (Fragment Per Kilobase per Million mapped reads) between the CapStarr-seq signal over the plasmid library (input). DHSs with a FC between 1.5 and 3 were labelled as ‘weak enhancer’ and DHSs with a FC equal or higher than 3 were labelled as ‘strong enhancer’. To visualize the CapStarr-seq signal per individual cloned fragments we generated a bed file with an RGB color code proportional to the enhancer activity.

### Conservation of *Ikzf1* enhancers

Mammalian conservation of *Ikzf1* enhancers and coordinates of human orthologues regions were assessed using the UCSC genome browser [48]. ChIP-seq data for H3K27ac in developing human thymocytes were obtained from the Blueprint consortium ([49]; http://dcc.blueprint-epigenome.eu; S3 Table).

### Analyses of ImmGen dataset

ATAC-Seq normalized signals for cell types of hematopoietic lineages were retrieved from the ImmGen project [50, 51] as bigwig files (GSE100738). Their coverage within IkE120 locus (chr11:11,564,323–11,564,574, mm10) was extracted using deepTools multiBigwigSummary (http://doi.org/10.1093/nar/gkw257) and compared to *Ikzf1* DeSeq2 normalized RNA-Seq signal of corresponding cell types obtained from the ImmGen portail (www.immgen.org).

### Hi-C and virtual 4C

Raw Hi-C data from primary DP thymocytes were taken from Hu et al. [52] and processed with FAN-C [53], entailing iterative mapping to the mm9 genome assembly with bowtie2, filtering self-ligation events and PCR duplicates, binning the data to 10 kb bins and balancing the chromosome-wide matrices with the Knight-Ruiz method. TAD boundaries were identified by computing insulation scores [54] with windows of 100 kb (10 bins), normalizing to chromosome-wide averages of insulation scores, then filtering the local minima with the delta vector calculated for the three bins flanking the computed one, and with the difference of the minima and maxima of the delta vector being at least 0.7. Virtual 4C plots were made by plotting the values for one “row” of the normalized Hi-C matrix (corresponding to the interactions of different bins with one specific bin, set to either the *Ikzf1* promoter or the E120 enhancer) against the genomic coordinate of the interacting bin.

### 4C-seq

Cell preparations were fixed with 2% formaldehyde in their respective culture medium for 10 min at 23°C. The fixation was quenched with cold glycine at a final concentration of 125 mM, then cells were washed with PBS and permeabilized on ice for 1 h with 10 mM Tris-HCl, pH 8, 100 mM NaCl, 0.1% NP-40 and protease inhibitors. Nuclei were resuspended in DpnII restriction buffer at 10 million nuclei/mL concentration, and 5 million nuclei aliquots were further permeabilized by treatment for either 1 h with 0.4% SDS at 37°C (ES cells), or for 20 min with 0.7% SDS at 65°C, then for 40 min at 37°C (DN3 and P5424 cells). The SDS was then neutralized by incubating for a further 1h with either 2.6% (ES) or 3.3% (DN3 and P5424) Triton-X100 at 37°C. Nuclei were digested overnight with 1000 U DpnII at 37°C, then washed twice by centrifuging and resuspending in T4 DNA ligase buffer. *In situ* ligation was performed in 400 μL T4 DNA ligase buffer with 20,000 U T4 DNA ligase overnight at 16°C. DNA was purified by reverse cross-linking with an overnight incubation at 65°C with proteinase K, followed by RNase A digestion, phenol/chloroform extraction and isopropanol precipitation. The DNA was digested with 5 U/μg Csp6I at 37°C overnight, then re-purified by phenol/chloroform extraction and isopropanol precipitation. The DNA was then circularized by ligation with 200 U/μg T4 DNA ligase under dilute conditions (5 ng/μL DNA), and purified by phenol/chloroform extraction and isopropanol precipitation. 50 ng aliquots of this DNA were used as template for PCR with a bait-specific primer located 1.2 kb upstream of E1L (chr11: 11,583,929–11,583,952) and containing Illumina adapter termini (optimal PCR conditions available on request). PCR reactions were pooled, primers removed by washing with 1.8x AMPure XP beads, then quantified on a Bioanalyzer (Agilent) before sequencing with a HiSeq 4000 (Illumina). All bait sequence (including and downstream of the primer sequence, up to but not including the GATC DpnII site) are trimmed by the demultiplexing Sabre tool (https://github.com/najoshi/sabre), allowing two mismatches, before mapping to the mm9 genome with Bowtie [55]. Mapped reads were processed and visualized by 4See [56]. Interactions were called by peakC [57] with a window size of 21.

## Results and discussion

### Prioritization of an *Ikzf1* enhancer

**Fig 1:**
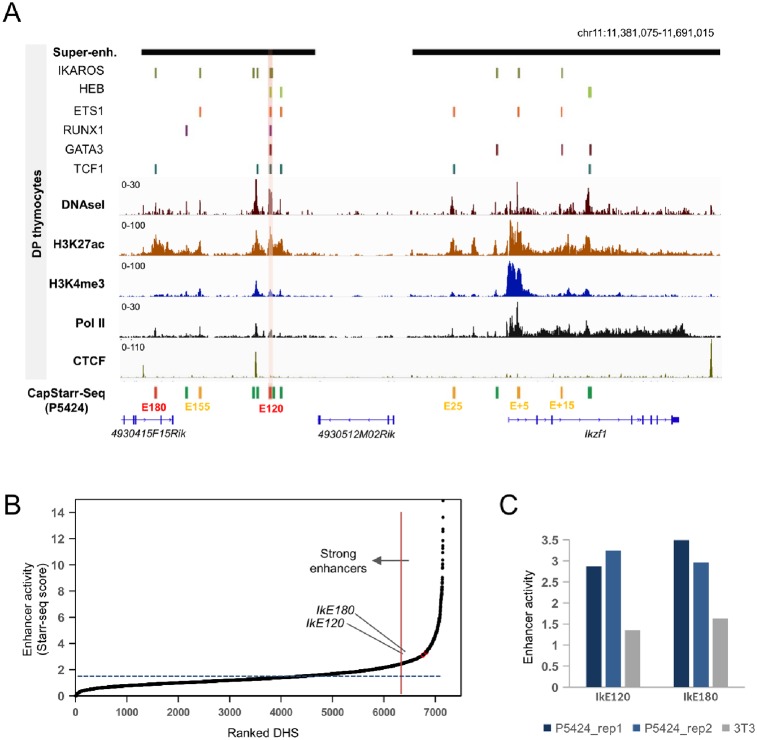
Prioritization of *Ikzf1* enhancers. (A) Epigenomic profiles of the *Ikzf1* locus showing ChIP-Seq signals for H3K4me1, H3K27ac and Pol II, DNAseI-seq, Super-enhancers and peaks of the indicated lymphoid transcription factors in mouse primary DP thymocytes. The enhancer activity of DHS regions as assessed by CapStarr-seq in P5424 cells (green: inactive; orange: weak; red: strong; merged from two replicates) is also shown. A strong enhancer associated with six transcription factors is highlighted. Coordinates of DHS and datasets are listed in S3 and S4 Tables, respectively. (B) Ranked DHSs from primary DP thymocytes in the function of enhancer activity assessed by CapStarr-seq in the P5424 cell line (merge of two replicates). The vertical line indicates the top 5% of the most active enhancer. The IkE120 enhancer is highlighted. (C) Enhancer activity of two *Ikzf1* enhancers assessed by CapStarr-seq in the P5424 and NIH3T3 cell lines.

In primary DP thymocytes, *Ikzf1* is associated with two clusters of enhancers or super-enhancers (Fig 1A). We identified thirteen DNAse I hypersensitive sites (DHSs) within the two super-enhancers in DP thymocytes (Fig 1A; S4 Table). To prioritize functional *Ikzf1* enhancers, we used our previously generated data from a CapStarr-seq high-throughput reporter assay [14] performed in the P5424 cell line [42], which roughly reflects T cell precursors [45]. In this assay, DHSs from primary DP thymocytes were assessed for enhancer activity [14]. Enhancer activity was calculated by the fold change (FC) of CapStarr-seq over the input signals for each DHS. As defined previously [14], active enhancers were classified as weak (1.5 < FC < 3) or strong (FC > 3). We found that 6 out of 13 *Ikzf1* associated DHSs displayed significant enhancer activity (Fig 1A). Of these, the weak enhancer located 15 kb downstream of the *Ikzf1* promoter (IkE+15) overlapped with a previously described enhancer [39, 41]. The two strongest enhancers (average FC higher than 3) were located 180 kb (hereafter IkE180) and 120 kb (hereafter IkE120) upstream of *Ikzf1* and have not been previously identified. These two strong enhancers overlapped the *Ikzf1* upstream super-enhancer and were classified within the top 5% of the most active DHSs present in primary DP thymocytes (ranked 360 and 405 out of 7,152 tested DHSs) (Fig 1B). Neither of the two enhancers was active in the fibroblast-derived NIH-3T3 cell line (Fig 1C). Analyses of H3K27ac ChIP-seq data from human developing thymocytes [58], suggested that the orthologous regions of IkE180 and IkE120 enhancers are also actives at certain stages of thymic T cell differentiation (S1 Fig).

We next explored whether the putative enhancers were bound by lymphoid specific transcription factors in primary DP thymocytes, using previously published ChIP-seq data for six transcription factors [14, 22, 59–61](S3 Table). Strikingly, the IkE120 enhancer was the only one to be bound by all tested lymphoid transcription factors, including Ikaros itself (Fig 1A). Interestingly, it was also located between two CTCF sites flanking the *Ikzf1* locus (Fig 1A), a known hallmark of “architectural” chromatin loops [62].

**Fig 2:**
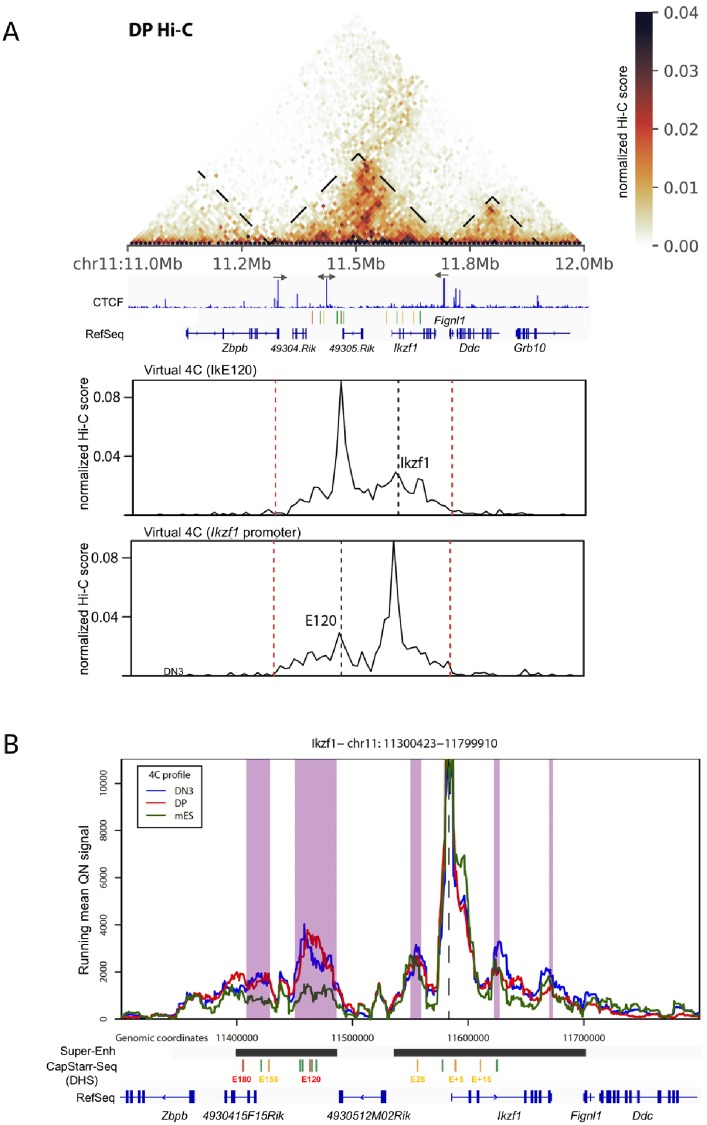
3D topology of the *Ikzf1* locus. (A) Hi-C view of primary DP thymocytes around the *Ikzf1* locus (top panel). TAD boundaries are shown. The orientation of the main CTCF peaks in primary DP thymocytes is displayed. Virtual 4C plots corresponding to the Hi-C interactions with the *Ikzf1* promoter or the E120 enhancer are shown in the bottom panels. (B) 4C-seq analysis of *Ikzf1* promoter interactions. Running mean (window of 21 fragments), quantile normalized 4C-seq profiles are shown from the *Ikzf1* promoter bait (dotted line) for primary DN3 (blue) and DP (red) thymocytes and mouse ES cells (green). Locations of genes and the six regions with enhancer activity in CapStarr-seq are shown below the plot. Conserved called interactions with thymic cells are highlighted in purple.

To assess whether the identified enhancers directly interact with the *Ikzf1* promoter, we initially analyzed published Hi-C data from primary DP thymocytes [52]. Identified enhancers were all embedded in the same Topological Associated Domain (TAD) as the *Ikzf1* locus, and flanked by convergent CTCF sites (Fig 2A). Virtual circularized chromosome conformation capture (4C) plots suggested that IkE120 and *Ikzf1* promoter preferentially interact together (Fig 2A, bottom panels). To directly demonstrate the interaction between IkE120 and *Ikzf1* promoter we analyzed published 4C-sequencing (4C-seq) experiments in primary DP thymocytes [56] along with newly generated 4C-seq data in primary CD44^+^CD25^+^ DN thymocytes (DN3) and non-expressing embryonic stem cells (ES), using the *Ikzf1* promoter as the viewpoint (Fig 2B). The *Ikzf1* promoter specifically interacted with the upstream super-enhancer in thymic cells but not ES cells, and displayed a strong interaction with the IkE120 enhancer.

To further interrogate how IkE120 enhancer relates to *Ikzf1* expression, we compared *Ikzf1* expression (RNA-seq) and chromatin accessibility (ATAC) at IkE120 enhancer using a comprehensive resource of hematopoietic cells from the ImmGen consortium [50, 51] (S2A Fig; representative examples are shown in S2B Fig). The IkE120 enhancer displayed the highest ATAC-seq signal in a subset of hematopoietic cells expressing moderated levels of *Ikzf1* expression, including T and B cell precursors and hematopoietic stem cells (HSC). In contrast, hematopoietic cells expressing high levels of *Ikzf1*, such as NK, γδ and mature CD4+ T cells, displayed a weak ATAC-seq signal at IkE120. Stroma cells that did not express *Ikzf1* were not associated with ATAC-seq peak at IkE120. These observations suggest that the IkE120 enhancer might play a preferential role in the expression of the *Ikzf1* gene in lymphoid precursors.

In conclusion, the IkE120 enhancer, displayed one of the strongest enhancer activities within the *Ikzf1* locus, was found to be associated with key lymphoid transcription factors and directly interacted with the *Ikzf1* promoter. While the regulatory elements within the *Ikzf1*-overlapping super-enhancer have been extensively studied [39, 41], the upstream super-enhancer harboring the IkE120 enhancer has remained unexplored. We, therefore, decided to further explore the functional role of this enhancer within its endogenous context.

### Deletion of the *Ikzf1* enhancer IkE120

**Fig 3:**
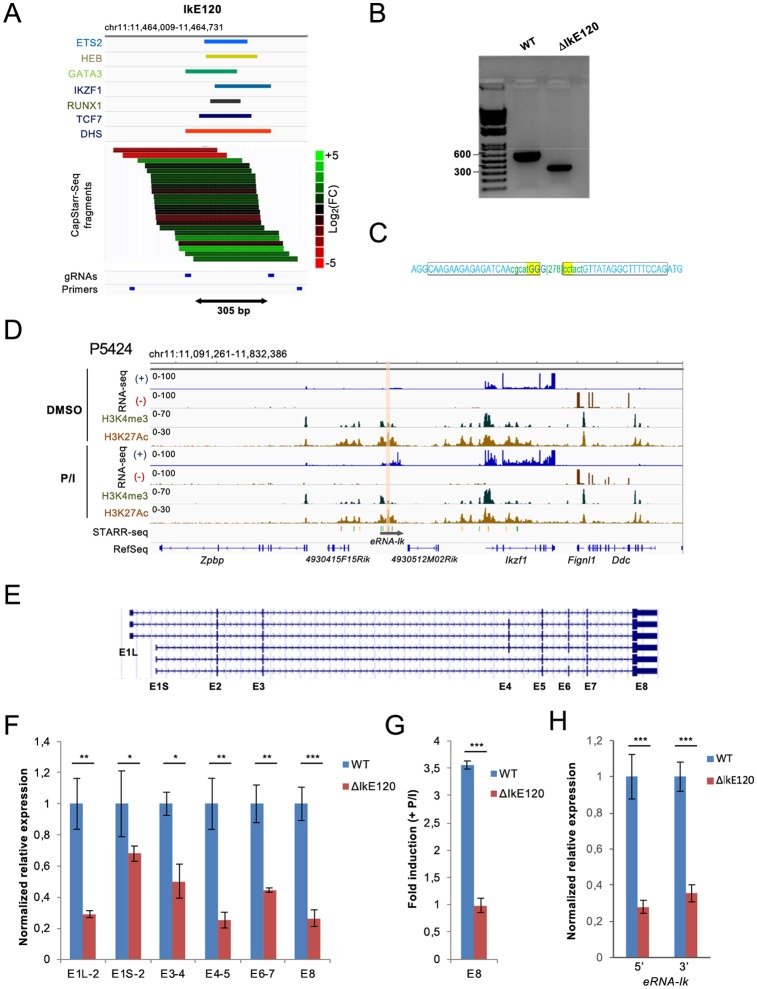
Deletion of the IkE120 enhancer. (A) Genomic tracks showing the binding peaks of the indicated transcription factors overlapping the IkE120 enhancer in primary DP thymocytes as well as the enhancer activity of individual clones assessed by the CapStarr-seq assay in P5424 cells. The color scale indicates the enhancer activity as a Log_2_ fold change of the CapStarr-seq signal over the input. The two sgRNAs used to delete the enhancer and primers to detect the deletion are also shown. (B) PCR analyses of IkE120 deletion in the P5424 cell line. (C) Sanger sequencing results from deletion junctions amplified from the genomic DNA of the targeted ΔIkE120 clone. The rectangles represent the position of the sgRNA. The deleted region is indicated in the bracket. (D) Genomic tracks for RNA-seq and ChIP-seq around the *Ikzf1* locus in P5424 cells stimulated or not with PMA/ionomycin (P/I). The IkE120 enhancer is highlighted. The scale of the RNA-seq tracks has been adjusted to visualize the non-coding transcripts overlapping the IkE120 enhancer (a screenshot with unmodified scales for the *Ikzf1* gene is shown in S3 Fig) (E) UCSC genome browser showing the transcripts isoforms of the *Ikzf1* gene found in RefSeq. (F) RT-qPCR analyses of *Ikzf1* expression at the indicated exon-exon junctions in wt and ΔIkE120 P5424 cells. Values represent relative expression as compared with wt samples. (G) Fold induction of *Ikzf1* expression (exon 8) after treatment with PMA and ionomycin of P5424 cells. (H) Relative expression of the non-coding transcripts (eRNA) overlapping the IkE120 enhancer in ΔIkE120 cells as compared with wt P5424 samples. Two sets of primers surrounding the IkE120 deleted region were used. In panels F-H, each point represents the means of three independent experiments normalized by the *Rpl32* housekeeping gene. Statistical significance was assessed by Student’s t-test (unpaired, two-tailed) from 3 biological replicates (***P < 0.001, **P < 0.01, *P < 0.1). Error bars represent standard deviation.

We used CRISPR/Cas9 technology to delete the IkE120 genomic region in the P5424 cell line, encompassing 305 bp covering the DHS site and the six transcription factor binding sites (ΔIkE120) (Fig 3A). Homozygous deletion of IkE120 was assessed by qualitative PCR and Sanger sequencing (Fig 3B and 3C). Note that the P5424 cell line was a valid model to study the endogenous IkE120 enhancer as the enhancer was highly enriched in H3K27ac in these cells and was associated with enhancer RNA (eRNA) expression (Fig 3D). In particular, expression of both *Ikzf1* and associated eRNA can be induced by the treatment of P5424 cells with PMA/ionomycin, which partially mimics T cell differentiation and β-selection [45](Fig 3D and S3 Fig).

Based on RefSeq annotation, the *Ikzf1* locus harbors 6 transcript isoforms (Fig 3E), which might play different regulatory functions [34–38]. We assessed the effect of Ik120 deletion on different exon-exon junctions encompassing all annotated *Ikzf1* transcripts by reverse transcription quantitative PCR (RT-qPCR) analyses in wild-type (wt) and ΔIkE120 P5424 cells (Fig 3F). The expression of the common 3’ UTR Exon 8 (E8) was decreased four-fold in the ΔIkE120 clone with respect to wt cells. Same results were observed for transcripts encompassing exons E4-E5, while those encompassing E3-E4 and E6-E7 were decreased only two-fold in the ΔIkE120 cells (Fig 3F). We also assessed promoter usages by quantifying the transcripts initiating from either E1L or E1S (Fig 3F). Transcripts originating from both promoters were significantly reduced, although the most upstream promoter appeared to be more affected (Fig 3F).

The deletion of the Ik120 enhancer completely inhibited the upregulation of *Ikzf1* by PMA/ionomycin treatment (Fig 3G). The Ik120 enhancer is associated with an eRNA transcript (hereafter eRNA-Ik), whose expression is correlated with *Ikzf1* induction in P5424 cells and during the DN to DP transition [45](Fig 3D). As expected, the expression of the eRNA-Ik transcript was strongly reduced in ΔIkE120 cells (Fig 3H).

In conclusion, the Ik120 enhancer appears to similarly regulate the different *Ikzf1* isoforms and is particularly required for the induction of the *Ikzf1* gene after cell stimulation.

### Deletion of IkE120 affects local epigenomic profiles

**Fig 4:**
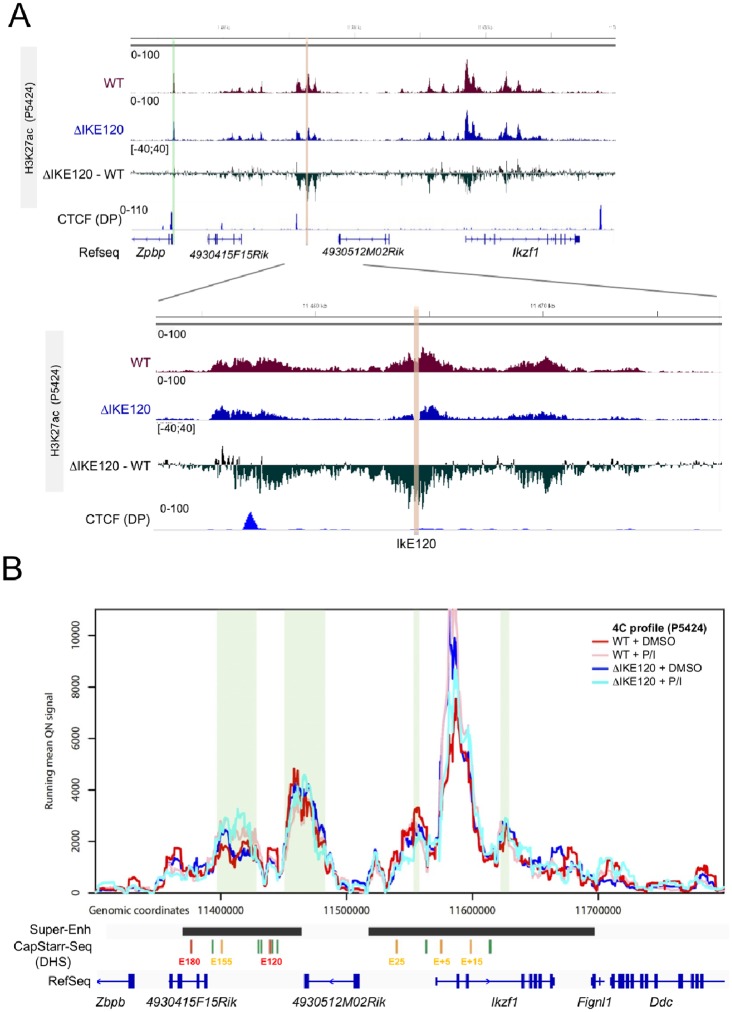
Epigenomic impact of IkE120 deletion. (A) The H3K27ac ChIP-seq at the *Ikzf1* locus (top) and around the IkE120 enhancer (bottom) in wt and ΔIkE120 P5424 cells are shown as individual tracks and as the differential signal between wt and ΔIkE120 cells. The genomic track of CTCF ChIP-seq in primary DP thymocytes is also shown. H3K27ac The IkE120 deleted region is highlighted in red and the promoter region of the neighbor *Zpbp* gene is highlighted in green. (B) Running mean (window of 21 fragments), quantile normalized 4C-seq profiles are shown from the *Ikzf1* promoter bait for wt unstimulated (red), wt stimulated (pink), ΔIkE120 unstimulated (blue) and ΔIkE120 stimulated P5424 cells. Locations of genes and the six regions with enhancer activity in CapStarr-seq are shown below the plot. Conserved called interactions are highlighted in green.

To assess whether IkE120 deletion affects the epigenomic profile of the *Ikzf1* locus we performed ChIP-seq experiments to assess H3K27ac profiles. As shown in Fig 4A, the deletion of IkE120 resulted in decreased levels of H3K27ac around the deleted enhancer region and to a lesser extent around the *Ikzf1* promoter, while H3K27ac at the promoter of the neighbor *Zpbp* gene was not affected. Besides, IkE120 deletion did not result in global changes of H3K27ac at gene promoters (S4 Fig). We next performed 4C-seq experiments using the *Ikzf1* promoter as a viewpoint in wt and ΔIkE120 P5424 cells in normal and stimulated conditions (Fig 4B). The genomic interactions observed in wt and mutant P5424 cells were very similar to the interactions observed in primary thymocytes (see Fig 2). Furthermore, no differences were observed between the wt and mutant cells, suggesting that IkE120 is not absolutely required for the establishment of the genomic interaction between the 5’ super-enhancer and the *Ikzf1* promoter (Fig 4B). Curiously, the promoter-IkE120 interaction is equivalent in normal and stimulated P5424 cells, whereas the interaction with IkE180 appears to increase slightly on *Ikzf1* upregulation during stimulation. Such findings are consistent with previous studies suggesting that some promoter-enhancer interactions are concomitant with transcriptional induction whereas others are formed prior to gene expression regulation [63]. Despite an overall reduction in *Ikzf1* expression and a complete loss of response to stimulation on IkE120 deletion, these topology dynamics are unchanged by the deletion. Overall, the IkE120 enhancer does not have a wide-spread influence on H3K27 acetylation and 3D topology of the locus, but rather contribute to localized epigenetic marking. This suggest that others regulatory elements within the 5’ super-enhancer are required to ensure the interaction with the *Ikzf1* promoters. Such role might be played by the DHS bound by CTCF upstream of the IkE120 enhancer (Figs 1A and 2A), which also corresponds to the 5’ border of influence of IkE120 on H3K27ac (Fig 4A).

## Conclusion

Integrative analyses of high-throughput reporter assays, chromatin structure and, 3D topology identified a strong enhancer (IkE120) associated with the *Ikzf1* gene. The deletion of the IkE120 enhancer using CRISPR/Cas9 technology demonstrated a critical role of this enhancer in controlling the expression of the *Ikzf1* gene. However, the IkE120 enhancer has a modest impact on the chromatin structure and 3D topology of the locus, highlighting the complexity of the regulatory landscape regulating the *Ikzf1* locus.

## Supporting information

S1 Fig A) Conservation of Ikzf1 enhancers across mammalian species. Detailed view of the IkE120 enhancer conservation is indicated at the bottom panel. B) H3K27ac tracks at the indicated human T cell precursors and Hematopoietic Stem Cells (HSC). The position of the human orthologous regions of the *Ikzf1* enhancers are indicated.
(PDF)

S2 Fig A) Comparison between *Ikzf1* expression and IkE120 chromatin opening in the hematopoietic lineages as indicated. Normalized RNA-seq and ATAC-seq data was retrieved from the ImmGen portal (http://www.immgen.org). B) ATAC-seq signal around the IkE120 enhancer (Left panel; signal scale was set to 5) and Ikezf1 expression (right panel) at selected hematopoietic samples.
(PDF)

S3 Fig Genomic tracks at the *Ikzf1* gene for the RNA-seq in P5424 cells stimulated or not with PMA/ionomycin(P/I). (PDF)

S4 Fig Average profiles and heatmaps of H3K27ac centered on the TSS of coding genes in wt and ΔIkE120 P5424 cells. (PDF)

S1 Table Primer sequences for CRISPR. (PDF)

S2 Table Primer sequences for RT-qPCR. (PDF)

S3 Table Information about published datasets used in this study and downloaded from the NCBI Gene Expression Omnibus. (PDF)

S4 Table List of DHSs associated with Ikzf1. The enhancer activity as assessed by CapStarr-seq in the P5424 cell line is indicated.
(PDF)

S1 Raw images Original gel image corresponding to Fig 3B. Lanes not included in the final figure were marked with an “X”. TrackIt 1 Kb Plus DNA Ladder (Thermo Fisher) was used as DNA ladder.
(PDF)
