# Correction: Integration of high-throughput reporter assays identify a critical enhancer of the *Ikzf1* gene

**DOI:** 10.1371/journal.pone.0235578

**Published:** 2020-06-25

**Authors:** The PLOS ONE Staff

## Notice of republication

An incorrect version of Fig 2 was published in error. This article was republished on June 16, 2020 to correct for this error. Please download this article again to view the correct version.
