# Supplementary figures and images for: Integration of high-throughput reporter assays identify a critical enhancer of the Ikzf1 gene
Source: PLoS One. 2020 May 26;15(5):e0233191. doi: 10.1371/journal.pone.0233191 (PMC7250416; doi:10.1371/journal.pone.0233191)

A

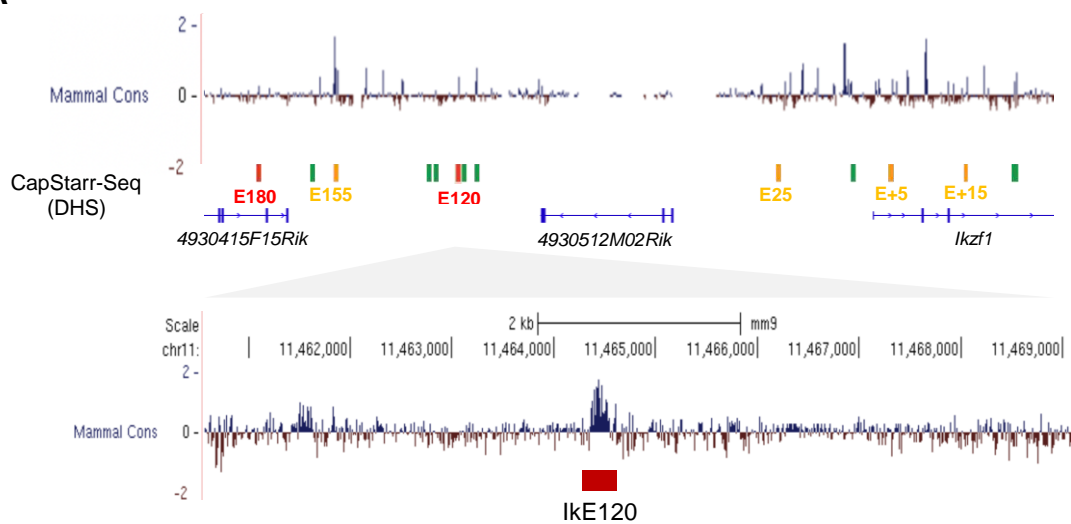

B

chr7:50,135,522-50,407,720 (H38)

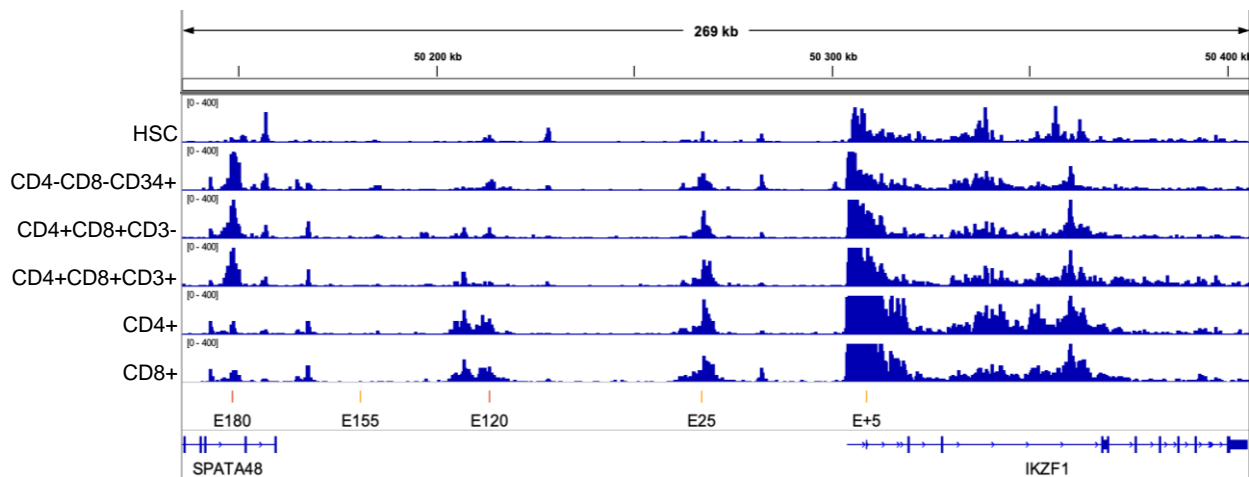

S1 Fig

Supplement: S1 Fig — A) Conservation of Ikzf1 enhancers across mammalian species. Detailed view of the IkE120 enhancer conservation is indicated at the bottom panel. B) H3K27ac tracks at the indicated human T cell precursors and Hematopoietic Stem Cells (HSC). The position of the human orthologous regions of the Ikzf1 enhancers are indicated. (PDF) [file pone.0233191.s001.pdf]

A

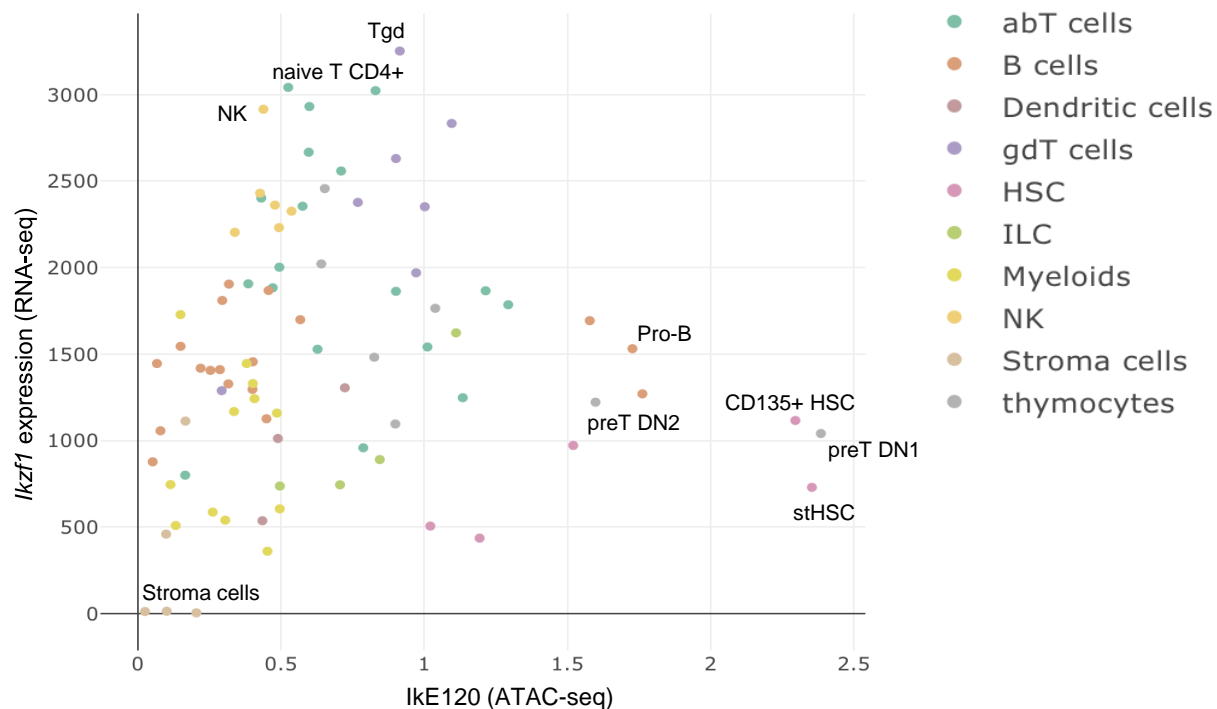

B

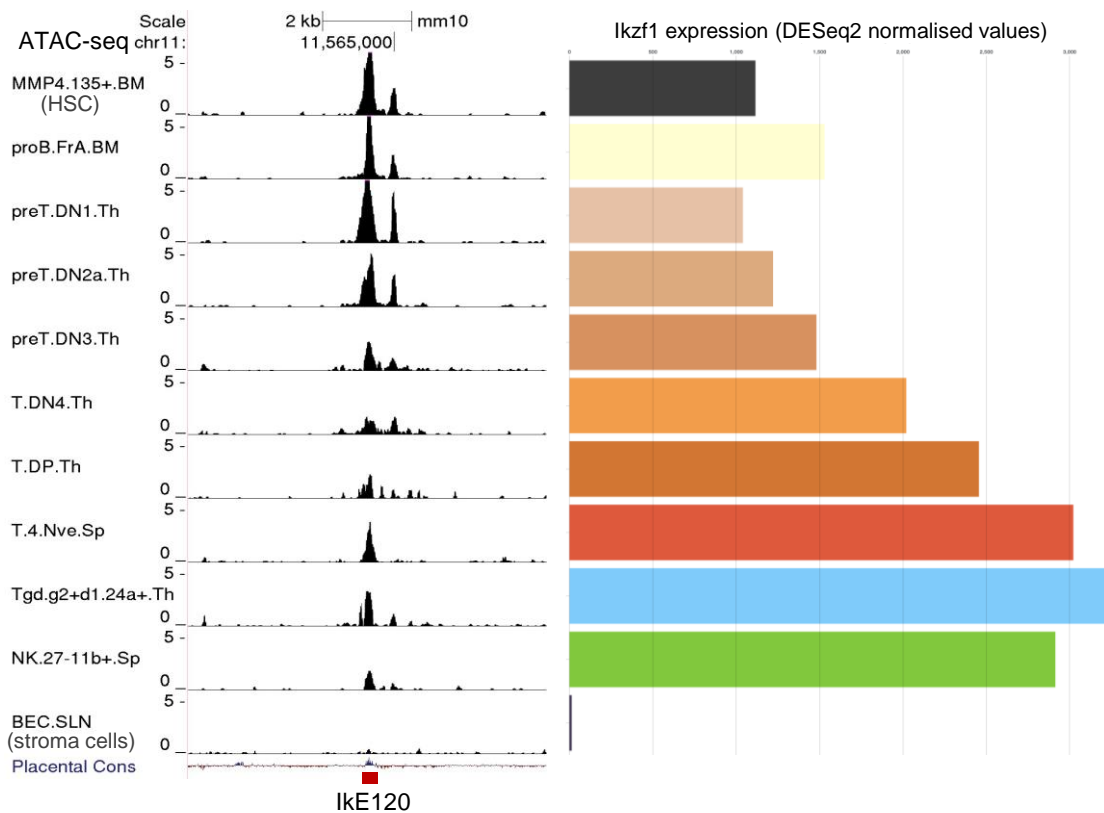

S2 Fig

Supplement: S2 Fig — A) Comparison between Ikzf1 expression and IkE120 chromatin opening in the hematopoietic lineages as indicated. Normalized RNA-seq and ATAC-seq data was retrieved from the ImmGen portal (http://www.immgen.org). B) ATAC-seq signal around the IkE120 enhancer (Left panel; signal scale was set to 5) and Ikezf1 expression (right panel) at selected hematopoietic samples. (PDF) [file pone.0233191.s002.pdf]

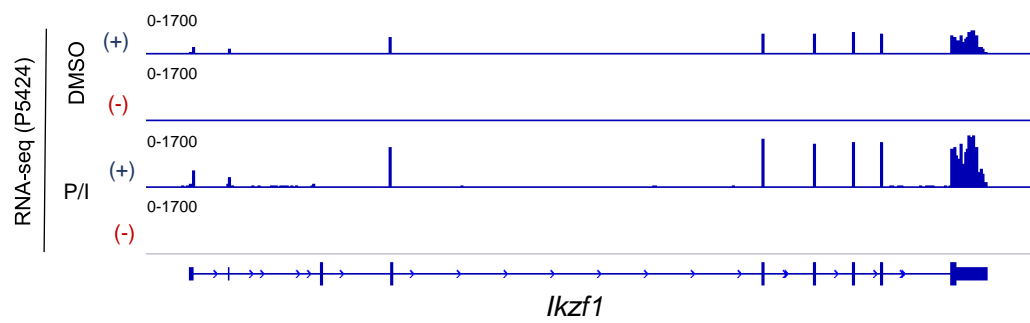

**S3 Fig**

Supplement: S3 Fig — (PDF) [file pone.0233191.s003.pdf]

**A**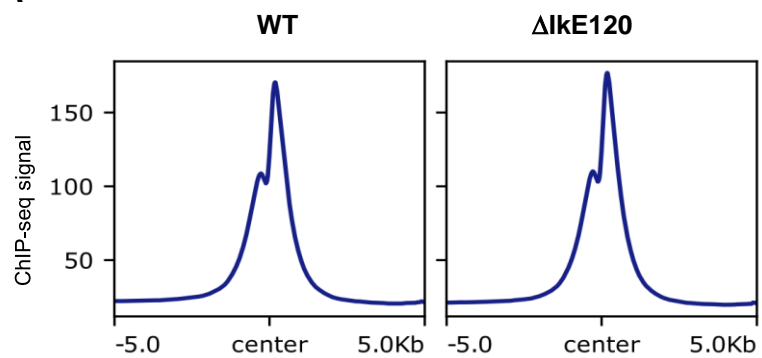**B**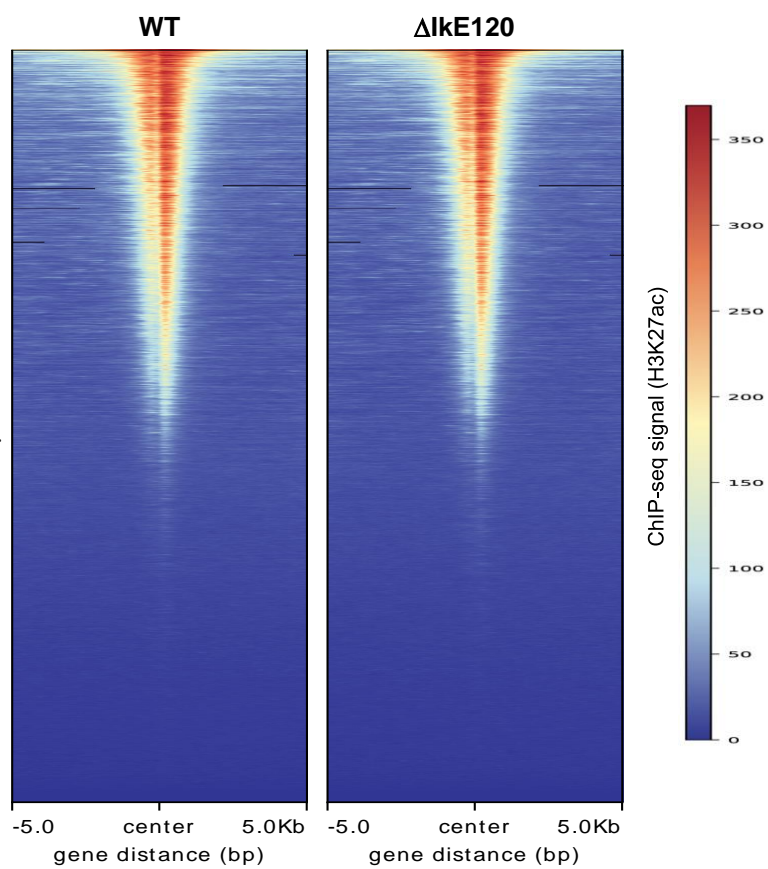**S4 Fig**

Supplement: S4 Fig — (PDF) [file pone.0233191.s004.pdf]

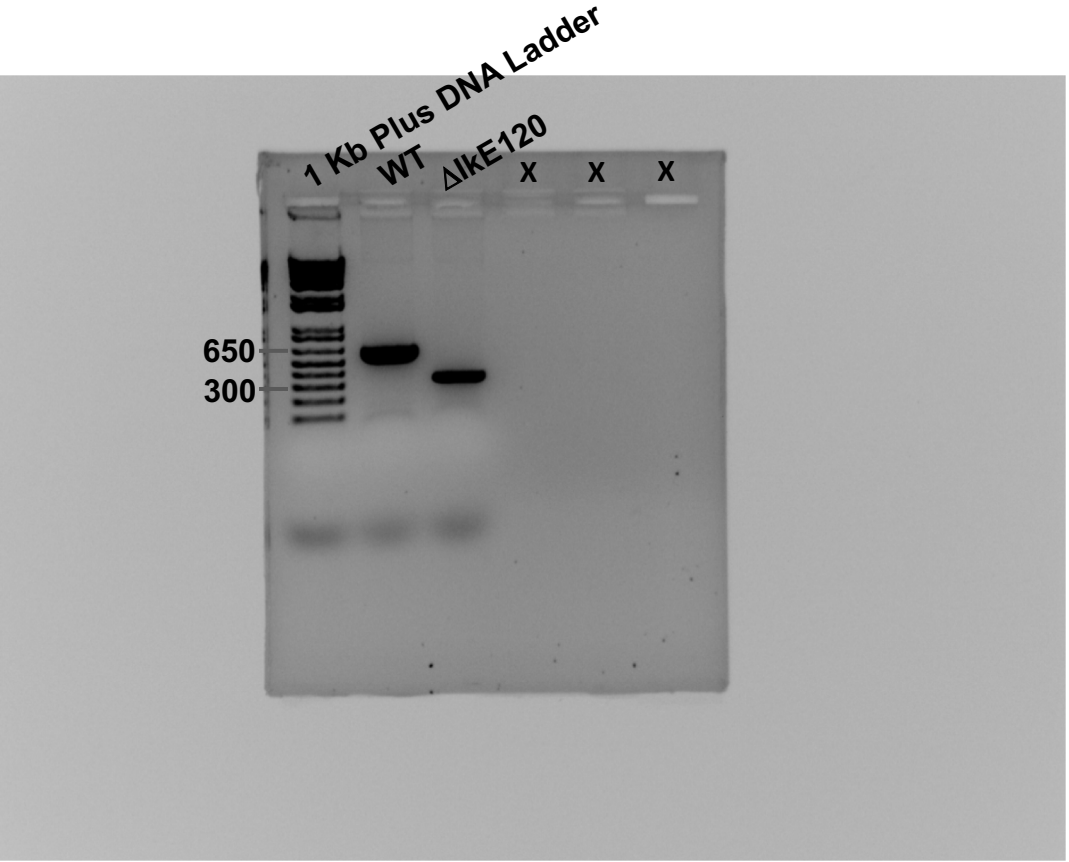

Supplement: S1 Raw images — Lanes not included in the final figure were marked with an “X”. TrackIt 1 Kb Plus DNA Ladder (Thermo Fisher) was used as DNA ladder. (PDF) [file pone.0233191.s009.pdf]
