# Supplementary material for: Integration of high-throughput reporter assays identify a critical enhancer of the Ikzf1 gene
Source: PLoS One. 2020 May 26;15(5):e0233191. doi: 10.1371/journal.pone.0233191 (PMC7250416; doi:10.1371/journal.pone.0233191)
Supplement: S1 Table — (PDF) [file pone.0233191.s005.pdf]

S3 Table. Primer sequences for CRISPR

Sequence (5' – 3')	Name
C-AAGAAGAGAGATCAACGCA	gRNA_Ikzf1_Upstream
ACTGTTATAGGCTTTTCCA-G	gRNA_Ikzf1_Downstream
GTTCAGGCAAATTTCAGAGG	Ikzf1_F
CTGGGAGGGTACTACTGCTC	Ikzf1_R
