# Supplementary material for: Integration of high-throughput reporter assays identify a critical enhancer of the Ikzf1 gene
Source: PLoS One. 2020 May 26;15(5):e0233191. doi: 10.1371/journal.pone.0233191 (PMC7250416; doi:10.1371/journal.pone.0233191)
Supplement: S2 Table — (PDF) [file pone.0233191.s006.pdf]

S4 Table. Primer sequences/ RT-qPCR

Sequences (5'- 3')-R	Sequences (5'- 3')-F	Name
GGGATTGGTGACTCTGATGG	GCTGCTGATGTGCAACAAA	RPL32
TTGACCCTCATCGACATCCA	CGCCCCAGGATCATTCTTG	IKZF1_E1L
TTGACCCTCATCGACATCCA	TTTGTGTGGCAGAGAGAGACA	IKZF1_E1S
GCTCATCCCCTTCATCTGGA	TGGATGTCGATGAGGGTCAA	IKZF1_E2-E3
CACACTGGTTGCACTGGAAA	ATCTGTGGGATCGTTTGCATC	IKZF1_E4-E5
CGCGCTGCTCCTCCTTGAGA	ACAGCGCAGCGGCCTTATCT	IKZF1_E8
TTCATGTCTTGCAACCCCTCA	TCCATTTCCCCTGCCATAGTTT	eRNA-Ik (5')
AGACACTGAGATGGGAAGGGA	CAAAGGGAGCTGGGGATGAG	eRNA-Ik (3')
