# Supplementary material for: Integration of high-throughput reporter assays identify a critical enhancer of the Ikzf1 gene
Source: PLoS One. 2020 May 26;15(5):e0233191. doi: 10.1371/journal.pone.0233191 (PMC7250416; doi:10.1371/journal.pone.0233191)
Supplement: S3 Table — (PDF) [file pone.0233191.s007.pdf]

S2 Table. Information about published datasets used in this study and downloaded from the NCBI Gene Expression Omnibus.

Sample	Reference	Source or GEO ID
CapStarr-seq in P5424 cells	[1]	GSE63732
RNA-seq from P5424 cells	[2]	GSE120655
ChIP-seq ETS1 from DP thymocytes	[3]	GSE29362
ChIP-seq Runx1 from DP thymocytes	[4]	GSE45014
ChIP-seq GATA3 from DP thymocytes	[5]	GSE20898
ChIP-seq Ikaros from DP thymocytes	[6]	GSE61148
ChIP-seq TCF1 from DP thymocytes	[1]	GSE63732
ChIP-seq HEB from DP thymocytes	[1]	GSE63732
ChIP-seq H3K27ac from DP thymocytes	[4]	GSE45014
ChIP-seq H3K4me3 from DP thymocytes	[3]	GSE29362
ChIP-seq Pol II from DP thymocytes	[3]	GSE29362
DNase I from DP thymocytes	[1]	GSE63732
ChIP-seq CTCF from DP thymocyte	[7]	GSE27214
4C-seq in DP thymocytes	[8]	GSE137417
ChIP-seq from human thymocytes (Blueprint consortium)	[9]	http://dcc.blueprint-epigenome.eu<br>Direct track hub:<br>http://genome-euro.ucsc.edu/cgi-bin/hgTracks?hubUrl=https://raw.githubusercontent.com/guillaumecharbonnier/mw-cieslak2019/master/src/hub/hub.txt&genome=hg19
Normalised RNA-seq data from mouse hematopoietic populations (ImmGen consortium)	[10]	www.immgen.org
Processed ATAC-seq data from mouse hematopoietic populations (ImmGen consortium)	[11]	GSE100738

Supplementary references

1. Vanhille L, Griffon A, Maqbool MA, Zacarias-Cabeza J, Dao LTM, Fernandez N, et al. High-throughput and quantitative assessment of enhancer activity in mammals by CapStarr-seq. Nat Commun. 2015;6:6905. doi: 10.1038/ncomms7905.
2. Saadi W, Kermezli Y, Dao LTM, Mathieu E, Santiago-Algarra D, Manosalva I, et al. A critical regulator of Bcl2 revealed by systematic transcript discovery of lncRNAs associated with T-cell differentiation. Scientific Reports. 2019;9(1):4707. doi: 10.1038/s41598-019-41247-5.
3. Koch F, Fenouil R, Gut M, Cauchy P, Albert TK, Zacarias-Cabeza J, et al. Transcription initiation platforms and GTF recruitment at tissue-specific enhancers and promoters. Nature structural & molecular biology. 2011;18(8):956-63. Epub 2011/07/19. doi: 10.1038/nsmb.2085. PubMed PMID: 21765417.
4. Lepoivre C, Belhocine M, Bergon A, Griffon A, Yammine M, Vanhille L, et al. Divergent transcription is associated with promoters of transcriptional regulators. BMC genomics. 2013;14:914. Epub 2013/12/25. doi: 10.1186/1471-2164-14-914. PubMed PMID: 24365181; PubMed Central PMCID: PMC3882496.
5. Wei G, Abraham BJ, Yagi R, Jothi R, Cui K, Sharma S, et al. Genome-wide analyses of transcription factor GATA3-mediated gene regulation in distinct T cell types. Immunity. 2011;35(2):299-311. Epub 2011/08/27. doi: 10.1016/j.immuni.2011.08.007. PubMed PMID: 21867929; PubMed Central PMCID: PMC3169184.
6. Oravecz A, Apostolov A, Polak K, Jost B, Le Gras S, Chan S, et al. Ikaros mediates gene silencing in T cells through Polycomb repressive complex 2. Nat Commun. 2015;6:8823. Epub 2015/11/10. doi: 10.1038/ncomms9823. PubMed PMID: 26549758; PubMed Central PMCID: PMC4667618.
7. Ebert A, McManus S, Tagoh H, Medvedovic J, Salvagiotto G, Novatchkova M, et al. The distal V(H) gene cluster of the Igh locus contains distinct regulatory elements with Pax5 transcription factor-dependent activity in pro-B cells. Immunity. 2011;34(2):175-87. Epub 2011/02/26. doi: 10.1016/j.immuni.2011.02.005. PubMed PMID: 21349430.
8. Ben Zouari Y, Platania A, Molitor AM, Sexton T. 4See: A Flexible Browser to Explore 4C Data. Front Genet. 2019;10:1372. Epub 2020/02/11. doi: 10.3389/fgene.2019.01372. PubMed PMID: 32038719; PubMed Central PMCID: PMCPMC6985583.
9. Stunnenberg HG, Hirst M. The International Human Epigenome Consortium: A Blueprint for Scientific Collaboration and Discovery. Cell. 2016;167(5):1145-9. Epub 2016/11/20. doi: 10.1016/j.cell.2016.11.007. PubMed PMID: 27863232.
10. Heng TS, Painter MW. The Immunological Genome Project: networks of gene expression in immune cells. Nature immunology. 2008;9(10):1091-4. Epub 2008/09/19. doi: 10.1038/ni1008-1091. PubMed PMID: 18800157.
11. Yoshida H, Lareau CA, Ramirez RN, Rose SA, Maier B, Wroblewska A, et al. The cis-Regulatory Atlas of the Mouse Immune System. Cell. 2019;176(4):897-912 e20. Epub 2019/01/29. doi: 10.1016/j.cell.2018.12.036. PubMed PMID: 30686579; PubMed Central PMCID: PMCPMC6785993.
