# Supplementary material for: Integration of high-throughput reporter assays identify a critical enhancer of the Ikzf1 gene
Source: PLoS One. 2020 May 26;15(5):e0233191. doi: 10.1371/journal.pone.0233191 (PMC7250416; doi:10.1371/journal.pone.0233191)
Supplement: S4 Table — The enhancer activity as assessed by CapStarr-seq in the P5424 cell line is indicated. (PDF) [file pone.0233191.s008.pdf]

S1 Table. List of DHSs associated with Ikzf1. The enhancer activity as assessed by CapStarr-seq in the P5424 cell line is indicated.

Coordiantes	Enhancer activity	Enhancer name
chr11:11406270-11406453	strong	E180
chr11:11421901-11422088	inactive	
chr11:11428864-11429017	weak	E155
chr11:11455715-11455824	inactive	
chr11:11457668-11457851	inactive	
chr11:11464295-11464600	strong	E120
chr11:11465199-11465308	inactive	
chr11:11469533-11469761	inactive	
chr11:11557248-11557441	weak	E25
chr11:11579095-11579318	inactive	
chr11:11589984-11590137	weak	E+5
chr11:11590190-11590299	weak	E+5
chr11:11611793-11612034	weak	E+15
chr11:11625705-11625906	inactive	
chr11:11625985-11626168	inactive	
chr11:11626256-11626493	inactive	
